# A Prestressing Strategy Enabled Synergistic Energy‐Dissipation in Impact‐Resistant Nacre‐Like Structures

**DOI:** 10.1002/advs.202104867

**Published:** 2022-01-12

**Authors:** Kaijin Wu, Yonghui Song, Xiao Zhang, Shuaishuai Zhang, Zhijun Zheng, Xinglong Gong, Linghui He, Hong‐Bin Yao, Yong Ni

**Affiliations:** ^1^ CAS Key Laboratory of Mechanical Behavior and Design of Materials Department of Modern Mechanics CAS Center for Excellence in Complex System Mechanics University of Science and Technology of China Hefei Anhui 230026 China; ^2^ Division of Nanomaterials and Chemistry Hefei National Laboratory for Physical Sciences at the Microscale Department of Chemistry Institute of Biomimetic Materials & Chemistry University of Science and Technology of China Hefei Anhui 230026 China

**Keywords:** biomimetic designs, impact resistance, nacre‐inspired separators, optimization strategies, prestressing strategies

## Abstract

The application of prestresses is a valuable strategy for enhancing the overall mechanical performances of structural materials. Residual stresses, acting as prestresses, exist naturally in biological structural materials, such as the nacre with the 3D “brick‐and‐mortar” arrangement. Although regulation of the tablets sliding has recently been demonstrated to be vital to improve toughness in synthetic nacre‐like structures, the effects of prestresses on the tablets‐sliding mechanism in these nacre‐like structures remain unclear. Here, by a combination of simulation, additive manufacturing, and drop tower testing the authors reveal that, at a critical prestress, synergistic effects between the prestress‐enhanced tablets sliding and prestress‐weakened structural integrality result in optimized impact resistance of nacre‐like structures. Furthermore, the prestressing strategy is easily implemented to a designed nacre‐inspired separator to enhance the impact resistance of lithium batteries. The findings demonstrate that the prestressing strategy combined with bioinspired architectures can be exploited for enhancing the impact resistance of engineering structural materials and energy storage devices.

## Graphical abstract

A bioinspired prestressing strategy is proposed to play the tablet‐sliding mechanism in nacre‐like structures more effectively. Synergistic effects between the prestress‐enhanced tablets sliding and prestress‐weakened structural integrality result in an optimized impact resistance at a critical prestress. The prestressing strategy is successfully implemented into the designed nacre‐like separators to significantly improve the impact tolerance and electrochemical performances of lithium batteries.

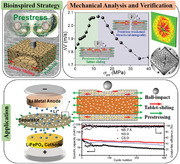

## 1 Introduction

**Figure 1:**
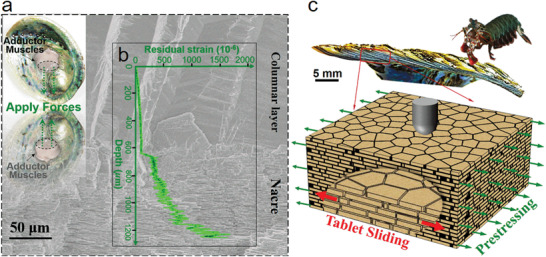
A prestressing strategy inspired from natural impact‐resistant nacre with residual stresses. a) The surface scanning electron microscope (SEM) images of the columnar and nacre layers in a fractured cross section of the bivalve mollusk shells (Inset shows that the adductor muscles can apply forces to close the shells of bivalve mollusks when they are attacked by predators). b) Experimental investigations of the internal residual strain release versus etched depth in mollusk shells, showing significant internal residual stresses in its gradient nacre layers, where the stresses arise from the gradient structural mismatching and the closing forces imposed by the adductor muscles. Reproduced with permission.^[^ ^22^ ^]^ Copyright 2009, Wiley‐VCH. c) Inspired from the survival war between the mantis shrimps and the mollusk shells with internal residual stresses in (b), an impact resistant model of the nacre‐like structure with prestresses was proposed to play the tablet‐sliding mechanism more effectively.

Biological structural materials often compose of the organic matter and minerals in multiscale hierarchically architectures,^[^ ^1^, ^2^, ^3^, ^4^, ^5^ ^]^ which can achieve mechanical performances far superior to their fragile constituents owing to multiplex toughening mechanisms.^[^ ^6^, ^7^, ^8^, ^9^, ^10^ ^]^ A representative toughening strategy relies on the internal residual stresses exiting in natural structural materials due to structural heterogeneities, chemical gradients, or service loads.^[^ ^11^, ^12^, ^13^ ^]^ The existence of the internal residual stress can be a prestressing strategy against forces impinging from outsides as well as affects dislocation motion and crack propagation, which in turn affects the toughness of biological structural materials.^[^ ^14^, ^15^, ^16^, ^17^ ^]^ A typical biological tough structure is the nacre from mollusk shells (**Figure** **1**a), which is built of microscopic mineral tablets and biopolymers in a 3D brick‐and‐mortar arrangement.^[^ ^18^, ^19^, ^20^ ^]^ Due to the structural mismatching between the organic and inorganic layers and the closing forces imposed by the adductor muscle, there exist significant internal residual stresses in nacre.^[^ ^21^, ^22^, ^23^ ^]^ Observations made by Wainwright show that residual stresses in mollusk shells are between 1.1 and 6.3 MPa, where stresses come from the closing forces imposed by the adductor muscles (Figure 1a).^[^ ^21^ ^]^ B. Pokroy et al.^[^ ^22^ ^]^ reported that the internal residual strains range from 0.01% to 0.15% across the shell thickness in the bivalve mollusk shells with gradient nacre layers (Figure 1b). However, above researches mainly focus on the distribution of internal residual stresses and their roles in biomineral morphologies or shell's shape for mollusk shells.^[^ ^13^, ^21^, ^22^, ^23^, ^24^ ^]^ The effects of internal residual stresses on the resistance of nacre to external loads remain to be explored at present.

In nature, mollusk shells with nacre layers often undergo concentrated impact loads normal to the surface of the shell, such as a dynamic strike by mantis shrimps.^[^ ^25^ ^]^ For the nacre with a brick‐and‐mortar arrangement under tensile or impact loads, millions of tablets can slide on one another over large volumes, along with large‐scale nonlinear shear deformations in interfaces.^[^ ^26^, ^27^, ^28^, ^29^, ^30^, ^31^ ^]^ This “tablet sliding mechanism” can significantly improve toughness, for example, the larger and more homogenously distributed sliding at the interfaces enables outstanding impact resistance of nacre‐like structures.^[^ ^32^, ^33^, ^34^ ^]^ It's worth noting that the efficient implementation of tablet‐sliding toughening mechanisms requires stringent control over numerous material and architectural parameters of nacre‐like structure,^[^ ^32^, ^33^ ^]^ and how to play the toughening roles of tablet‐sliding in nacre‐like structure more simply and effectively still needs to be explored. Meanwhile, previous researches ignore the influences of internal residual stresses on the tablet sliding mechanism in nacre. For engineering structural materials composed of hard and soft phases, such as concrete or composite laminates, initial internal stresses have been widely used as a prestressing strategy to enhance impact resistance, where the prestresses can be produced either by service loads or by the manufacturing process.^[^ ^35^, ^36^, ^37^ ^]^ Previous analytical investigations show that the tensile prestresses can elevate the maximum contact forces as well as reduce the contact time and the deflection.^[^ ^38^, ^39^ ^]^ J. Suhr et al. reported that prestresses can significantly improve the effectiveness of the nanotube‐sliding to enhance dynamic damping in carbon nanotube polymer composites.^[^ ^40^, ^41^ ^]^ Inspired by above results, we speculate that the prestressing strategy and tablet‐sliding mechanism can work synergistically to further enhance the impact resistance of nacre‐like structures.

To prove above assumption, inspired by the natural phenomenon that the bivalve mollusk shells with internal residual stresses can withstand the dynamic strike of mantis shrimps,^[^ ^42^ ^]^ where the adductor muscles in bivalve mollusks can apply preforces to close the shells when they are attacked by predators,^[^ ^21^ ^]^ we constructed a 3D model for the nacre‐like structure with prestresses under impact loading (Figure 1c). Nonlinear finite element modeling simulations revealed that nacre‐like structures with prestress can achieve higher impact resistance (energy dissipation during impaction), where the prestress can promote tablets sliding. Meanwhile, at a critical prestress, synergistic effects between the prestress‐enhanced tablet‐sliding and prestress‐weakened structural integrality result in maximum impact resistance. Drop tower tests on 3D‐printed nacre‐like specimens under pretensioning proved the critical prestress corresponding to optimized impact resistance. At last, inspired from the toughening mechanisms in nacre, we designed a nacre‐inspired separator (NS),^[^ ^43^ ^]^ and the prestressing strategy was easily implemented to it to effectively enhance the impact tolerance of lithium batteries.

## 2 Results and Discussion

### 2.1 A Prestress‐Toughening Strategy for Impact‐Resistant Nacre‐Like Structures

We carried out dynamic finite element simulations to explore the effects of prestress on the impact‐resistance of nacre‐like structure. In simulations, we developed a 3D nonlinear finite element model for the nacre‐like structure that duplicates the “brick‐and‐mortar” arrangement of nacre from mollusk shells (Figure S1, Supporting Information). A prestress field was imposed on the nacre‐like structures by predefining stress field in ABAQUS before impact simulations, which mimics the scenario that internal residual stresses exist in nacre layers of mollusk shells due to the structural mismatching and the closing forces imposed by the adductor muscle.^[^ ^21^, ^22^ ^]^ We focus on the synergistic effects between the prestressing strategy and the tablet sliding mechanism in nacre‐like structures, where the tablet sliding can generate nonlinear deformation over large volumes and is key to the impact resistance of natural nacre.^[^ ^32^, ^33^ ^]^ It's worth mentioning that the absolute values of the impact performances simulated here differ from that of natural nacre since the highly idealized model we used adopts different size and geometry, but the model replicates the “brick‐and‐mortar” structural features quite well and can provide insights into the key role of the prestress on the impact resistance of nacre‐like structure.

**Figure 2:**
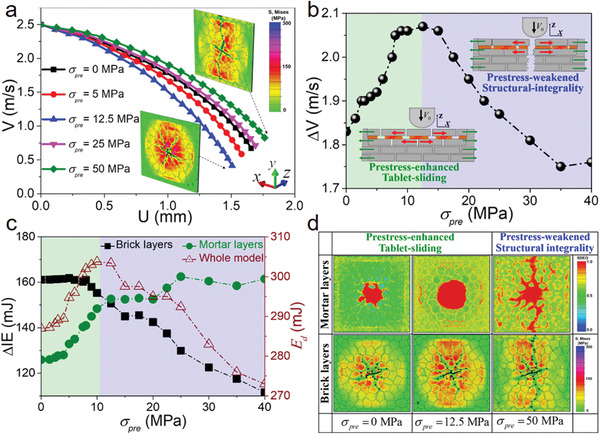
Effects of the prestresses on the impact‐resistance of the nacre‐like structure. a) Residual velocity (*V*)–displacement (*U*) curves for the nacre‐like structure under different prestresses (*σ*_pre_), and the insets of the von Mises stress fields show the damage patterns under different *σ*_pre_. b) Plot of the impact speed loss (ΔV) under different *σ*_pre_, and the insets show the schematics of failure modes under different *σ*_pre_. c) The internal energy dissipations ΔIE in mortar layers and brick layers and the energy dissipation (*E*_d_) of whole model under different *σ*_pre_. d) Maps of the sliding deformation in the lowermost mortar layer and the failure patterns (von Mises stress field) in the back brick layer under different *σ*_pre_, where SDEG (scalar stiffness degradation variable field) = 0 represents zero failure while SDEG = 1 stands for the complete failure.

**Figure** **2**a shows the impact velocity–displacement curves of nacre‐like structure under different prestresses. The results indicate that with the increase of prestress, the attenuation of impact velocity presents a trend of first intensifying and then slowing down within a same time interval. Meanwhile, the simulation insets in Figure 2a show the von Mises stress profiles and failure modes at last impact moment for nacre‐like structures under different values of prestress. It is clear from the simulation images that for nacre‐like structures with a low prestress of 12.5 MPa, there is a wide stresses distribution underneath the projectile, and numerous radial cracks propagate through the hard and soft materials by following tortuous paths over enlarged contact regions. This phenomenon originates from the hard‐soft staggered architecture of the nacre‐like design and also represents the fact that, upon impact, a large number of hard tablets can slide on one another over large volumes, which results in large amounts of impact energy dissipations. It is noteworthy that, for the nacre‐like structure under an excessive prestress of 50 MPa, a through‐sample crack occurs and the structure breaks catastrophically into two parts upon impact, indicating limited impact‐resistance. Hence the initial prestress fields have important influences on the impact resistance of nacre‐like structure. Furthermore, a critical prestress at which the impact‐velocity loss reaches maximum can be found from the velocity loss versus prestress curve in Figure 2b. This phenomenon indicates that the impact‐resistance of nacre‐like structures can be optimized through tuning the values of prestress. Meanwhile, the values of the critical prestress increase as the interfacial strength increases (Figure S2a, Supporting Information). For the nacre‐like structure with the interfacial strength of 25 MPa, the simulated value of critical prestress is about 12.5 MPa (Figure 2b), which is in good agreement with the values of internal residual stresses in nacre (with a modulus of about 60 GPa) from mollusk shells, where the internal residual stresses range from 6 to 90 MPa due to the gradient structural mismatching and the closing forces imposed by the adductor muscles in mollusk shells.^[^ ^21^, ^22^ ^]^

To reveal the underlying mechanisms for the existence of critical prestress, we analyzed the energy dissipations of bricks layers and soft mortar layers, respectively, in the nacre‐like structures under different prestresses (Figure 2c). Results indicate that with the increase of prestress, the energy dissipations in brick layers initially stay flat and then decrease sharply, whereas the energy dissipations in mortar layers first rise sharply and then tend to be constant. At a critical prestress, the energy dissipations in brick layers and mortar layers become approximately equal and the whole energy dissipation of the nacre‐like structure is maximum (Figure 2c). In addition, for the nacre‐like structures with different interfacial strength, the intersection of energy dissipation–prestress curves of brick layers and mortar layers also corresponds to the critical stress at which the nacre‐like structure shows optimal impact resistance (Figure S2b, Supporting Information). Therefore the critical prestress results from the compromise in competition of energy dissipations between the shear deformations (sliding and delamination, etc.) at mortar layers and tensile deformations (crack propagation) at brick layers. Plots of the von Mises stress profiles in the back brick layer and the scalar stiffness degradation fields (SDEG) in the lowermost mortar layer provided comprehensive pictures for the deformation mechanisms in nacre‐likes structures under different prestresses (Figure 2d). We were particularly interested in revealing the effects of prestress on the tablets sliding, which is the main mechanism for energy dissipation in nacre‐like structures. The SDEG quantifies the amount of interlayer deformations triggered by tablets sliding, where SDEG = 0 (blue color) represents no tablets sliding failure and SDEG = 1 (red color) represents complete delamination failure caused by excessive tablets sliding. In the nacre‐like structures without prestress, the SDEG in the lowermost mortar layers show relatively small and localized tablets sliding, and the characteristic radial cracks occur in brick layers by following torturous paths. With the increase of prestress, the areas of tablets sliding and delamination are enlarged and the radial cracks spread out more in the nacre‐like structure with a critical prestress, exhibiting a wider stresses distribution underneath the impactor. When the prestress is so large that exceeds a critical value, the structural integrality of nacre‐like structures was significantly weakened with a through‐sample crack formed due to excessive tablets sliding caused by prestress. This phenomenon indicates the fact that due to excessive prestress, limited energy dissipations caused by interlayer sliding and intralayer deformations take responsibility for impact‐resistance of nacre‐like structures. Thus, there exist two dominated failure modes for energy dissipation in nacre‐like structures with prestress, including the prestress‐enhanced tablets sliding under low prestress and the prestress‐weakened structural integrality at excessive prestress (Figure 2d and insets of Figure 2b). At a critical prestress, synergistic effects between the two energy‐dissipation modes result in optimized impact resistance of nacre‐like structures.

### 2.2 Experimental Verifications for the Prestressing Strategy

**Figure 3:**
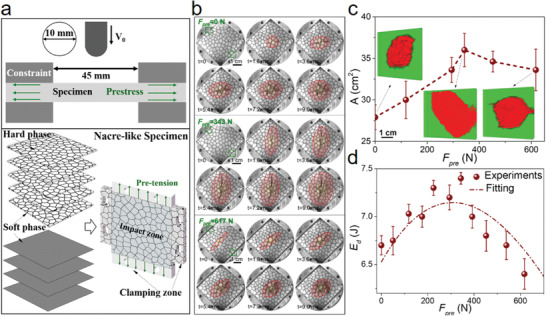
Impact tests of the 3D‐printed nacre‐like structures with prestresses. a) Experimental setup for the drop‐tower tests of the 3D‐printed nacre‐like structures with prestresses, and the geometry of the nacre‐like specimen in 3D printing. b) Evolution of the failure morphologies on impacted 3D‐printed nacre‐like structures under different pretensile forces (*F*_pre_), where the red shadows represent crack propagation zones. c) The contour area (A) and morphologies for the failure zones (red color) of the impacted specimens under different *F*_pre_. d) Impact energy dissipation *E*_d_–*F*_pre_ curve for the 3D‐printed nacre‐like specimens.

We further performed droptower tests on 3D‐printed nacre‐like structure with prestress to verify the effects of prestress on impact‐resistance. **Figure** **3**a shows the experimental setups and the geometry of 3D‐printed nacre‐like specimens. The nacre‐like specimens composed of hard phases (VeroWhitePlus) and soft phases (TangoblackPlus) with brick‐and‐mortar arrangements mimicking the natural nacre architecture were fabricated using multimaterials 3D‐pringting technology (Figures S3 and S4, Supporting Information). The clamping zones printed at the same time as nacre‐like specimens were used for applying prestress. The droptower test machine equipped with a high speed camera and a designed pretensile device was used to perform impact tests on 3D‐printed nacre‐like specimens with prestress (Figure S5, Supporting Information). The prestress fields were imposed on the 3D‐printed nacre‐like samples by applying pretensile forces before droptower tests. Details of fabrication and impact tests for 3D‐printed nacre‐like specimens can be seen in Supporting Information.

First, we focus on the effects of prestress on failures modes of nacre‐like structure under impact. For the nacre‐like specimens under different values of pretensile forces, the failure patterns observed from the surface opposite the impact direction are compared, and the dynamic evolution of crack patterns is recorded in situ with a high speed camera (Figure 3b). For the specimens without pretensile force (*F*_pre_ = 0 N), cracks propagated through the hard bricks and soft mortars, and a pattern of radial cracks formed underneath impactor, showing tortuous crack paths, and localized microcracks formation. This phenomenon is due to the tablets sliding in nacre‐like structures and is in good agreement with the above simulated failure patterns. As *F*_pre_ increases, a radial cracks pattern composed of a main crack formed early and multiple radial subcracks spread out more over a wider deformation zone, and the impactor didn't result in complete perforation and bounced back. These delocalization deformations demonstrate that the entire nacre‐like structure in different sections works in concert to take the impact load. This is due to the larger and more homogenously distributed tablets sliding, which is enhanced by pretensile forces. When the *F*_pre_ is so large that exceeds the strength limits of the structure, a through‐sample crack forms at the beginning, then the spalling and perforation occur after impaction, indicating limited impact energy dissipation due to that excessive pretensile forces weaken structural integrity. Further, X‐ray computed tomography (X‐ray CT) of impacted specimens provided a comprehensive picture of the micromechanics of deformation inside the 3D‐printed nacre‐like specimens (Figure S6a, Supporting Information). Insets of Figure 3c highlight the morphologies for the interior failure zones composed of the intralayer cracks and interlayer delamination in the nacre‐like structures with different *F*_pre_. With *F*_pre_ increases, the contour area of failure zone becomes much larger (Figure 3c). When *F*_pre_ is so large that exceeds a critical value, a through‐sample crack appears and the area of failure zone decreases. Thus, above experimental results clearly reveal two dominated failure modes in nacre‐like structures with prestress, including the prestress‐enhanced tablets sliding and the prestress‐weakened structural integrality, which are in good agreement with simulation results.

Further, typical velocity–displacement curves for 3D‐printed nacre‐like specimens under different *F*_pre_ are shown in Figure S6b, Supporting Information. The results indicate that the appropriate *F*_pre_ can expedite the attenuation of impact velocity, and the impactor bounces back, whereas the impactor with residual velocity perforates the nacre‐like specimens with overlarge *F*_pre_. Meanwhile, the force–displacement curves for nacre‐like structures under different *F*_pre_ are shown in Figure S6c, Supporting Information. The results show that with the increase of *F*_pre_, the contact force and effective modulus first increase, and then significantly decrease at overlarge *F*_pre_, which is due to that the excessive pretension causes catastrophic through‐sample cracks and weakens structural integrity. The experimental results are in good agreement with previous theoretical results,^[^ ^38^, ^39^ ^]^ where the tensile prestress can elevate the maximum contact forces as well as reduce the deflection in composite structures under impact. In addition, the step‐like fluctuations in curves reveal the existence of tablets sliding and localized microcrack formation in the nacre‐like structures with brick‐and‐mortar arrangements. At last, Figure 3d shows the impact energy dissipations of nacre‐like specimens under different *F*_pre_, where there exists a critical value of *F*_pre_ at which the energy dissipation is maximum, that is, the impact‐resistance is optimized. Thus, above experimental results also reveal that at a critical prestress, synergistic effects between the prestress‐enhanced tablet sliding and prestress‐weakened structural integrality result in optimized impact resistance of nacre‐like structures. This experimental phenomenon is in good agreement with our simulated results, but absolute values of the critical prestress will vary with geometry and materials in experiments and simulations. Therefore, our findings provide a new insight that the prestressing strategy can work synergistically with the tablets sliding mechanisms to further enhance the impact resistance of nacre‐like structures.

### 2.3 Application of the Bioinspired Prestressing Strategy

**Figure 4:**
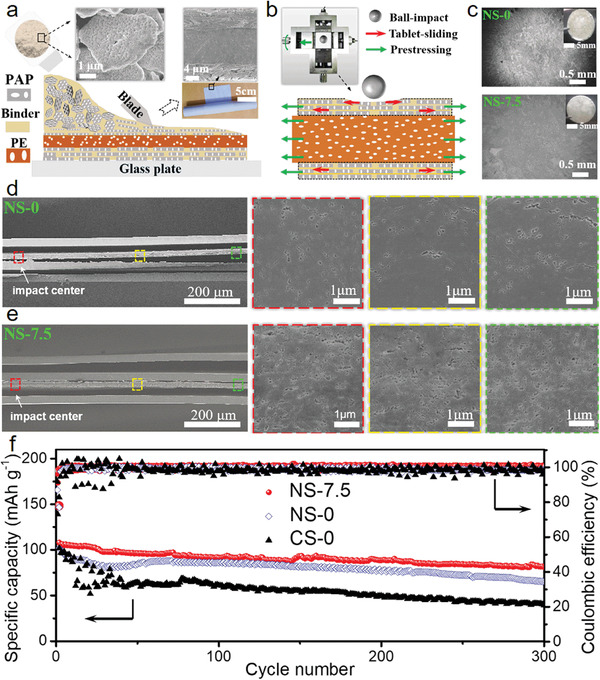
Prestresses enhance the impact resistance for a nacre‐inspired separator of lithium batteries. a) Schematic diagram for the design procedure of a nacre‐inspired separator (NS), which is composed of the porous aragonite platelets (PAP) coating with a “brick‐and‐mortar” arrangement and microporous polyolefin (PE) membranes. Left top insets are the PAP slurry and its SEM image, the right top insets are the photograph of as‐obtained NS and its cross‐sectional SEM image. b) Schematic impact‐resistance of NS under prestressing. Left top inset shows a special fixture for applying prestress. c) Top‐view SEM images of Li foil after Li plating using the impacted NS, where NS‐0 and NS‐7.5 represent the pretensile forces are 0 and 7.5 N, respectively. d,e) Overview of the cross‐sectional SEM image of the impacted NS‐0 (d) and NS‐7.5 (e), and different color squares correspond to the enlarged SEM images of different locations. f) Long‐term cycling of the LiFePO_4_/Li coin cells using the impacted NS‐7.5, NS‐0, and CS‐0, respectively, at 0.2 C for first five cycles and 2 C for the following cycling, where CS represents the commercial separator composed of ceramic nanoparticles coating and PE.

Inspired by the synergistic effects between the prestressing strategy and the tablets sliding mechanism in nacre‐like structures, we applied prestresses to our designed NS to further enhance the impact resistance of lithium batteries. The NS, which is composed of the porous aragonite platelets (PAP) coatings with a nacre‐like brick‐and‐mortar arrangement and the microporous polyolefin (PE) membrane, was constructed by a facile slurry coating method (**Figure** **4**a). Details of preparations and characterizations of the NS are provided in Supporting Information. The surface scanning electron microscope (SEM) image of the as‐fabricated NS shows that the PAP are highly horizontally aligned to form the nacre‐like multilayer structures on the surface of the PE membrane (insets of Figure 4a). To demonstrate the effectiveness of our proposed prestressing strategy, we carried out the ball impact tests on our developed nacre‐like separator under different prestresses (Figure 4b and Figure S7, Supporting Information), where the prestress fields were imposed on the nacre‐like separators by applying pretensile forces before droptower tests before ball impact tests. Based on above mechanical analyses, appropriate prestress can facilitate tablets sliding, and the localized impact force can be dispersed into wide and uniform stress distribution in the nacre‐like coatings of the NS. As a result, we consider that the pores inside the PE layer of NS with prestress can be prevented from closing and the Li^+^ ion flux in the separator remains homogeneous distribution. To verify this, the impacted separators were assembled into Li–Li symmetric cell to observe Li deposition morphologies. The Li–Li symmetric cell using the impacted nacre‐like separator with the pretensile force of 7.5 N (NS‐7.5) showed more homogeneous lithium deposition than that of the cell using the NS‐0 (Figure 4c), where the impacted NS‐7.5 can maintain nearly same amount of lithium deposited in the impact area as that in the normal area, indicating uniform Li^+^ ion flux. In contrast, for the commercial separators (CS) composed of ceramic nanoparticles coatings and a microporous PE membrane, the catastrophically perforated damage would occur and lots of pores close in PE under impaction (Figure S8a, Supporting Information). The Li–Li symmetric cell using the impacted CS without prestress (CS‐0) showed a very inhomogeneous Li plating due to nonuniform Li+ ion flux induced by localized close pores and cracks in the impact area (Figure S8b, Supporting Information). Further, the cross‐sectional SEM images of separators after impaction show detailed pore deformation inside the PE layers (Figure 4d,e). The impacted NS‐7.5 (Figure 4d) can still have more nanoscale pores inside the PE layers than that of the impacted NS‐0 (Figure 4e), demonstrating that the prestressing strategy can effectively enhance impact resistance of the nacre‐like separator. In contrast, lots of close pores and cracks were generated in the impact area of the CS‐0 (Figure S8c, Supporting Information), indicating limited energy dissipation. Further, nonlinear finite element modeling simulations show more comprehensive impact performances of separators under different prestresses (Figure S9, Supporting Information). The attenuation of impact velocity in NS‐7.5 is more obvious than that in NS‐0 and CS‐0 within same time intervals (Figure S9b, Supporting Information). The von Mises stress fields show that the stress distribution in PE layer of CS‐0 is highly localized and the catastrophically perforated failure occurs in nanoparticle coatings of CS‐0 (Figure S9c, Supporting Information). The stresses in PE layer of NS‐7.5 are smaller and more evenly distributed than that of NS‐0 and CS‐0, and the wider delocalization stress distribution occurs in nacre‐like coatings of NS‐7.5 due to tablets sliding. Moreover, the energy dissipation curves show that compared with the NS‐0 and CS‐0, the NS‐7.5 dissipates most of the impact energy at nacre‐like coatings and the energy dissipation in PE layer is lowest (Figure S9d, Supporting Information).

To demonstrate the effects of prestressing strategy on the electrochemical performances of the separator, we assembled and tested the LiFePO_4_/Li coin cells using the impacted NS‐7.5, NS‐0, and CS‐0. The cell using the impacted separators displayed typical charge/discharge voltage profiles at 2 C rate (Figure 4f). The cell using the impacted NS‐7.5 exhibited higher specific capacity and better cycling stability that the cells using the impacted NS‐0 and CS‐0 at the rate of 2 C. After the 300 cycles, the cell using the impacted NS‐7.5 remained a specific capacity of about 95 mAh g^−1^ much higher than that of the cells using the impacted NS‐0 (70 mAh g^−1^) and CS‐0 (35 mAh g^−1^). In addition, the cell using the impacted NS‐7.5 showed better cycling stability and Coulombic efficiency (≈99.8%) in contrast to the cell using the impacted NS‐0 and CS‐0. These findings demonstrated that the prestressing strategy can be easily implemented to a designed NS to enhance the impact resistance, thus maintain the homogenous distribution of Li^+^ ion flux in the cell to gain safety improvement of lithium batteries. It is worth mentioning that in practical production of lithium‐ion batteries,^[^ ^44^ ^]^ one idea is that we can control the relative speed of the winding shaft in the battery winding process, so that prestressing strategy can be effectively applied to our designed nacre‐like separators (Figure S10, Supporting Information). Meanwhile, considering the critical prestress corresponding to the optimal impact resistance, we need to carefully apply tensile preloads on nacre‐like structures to guarantee that no preloads‐induced damage was generated before impact and the energy‐dissipation ability of tablet sliding can be effectively utilized.

## 3 Conclusions

In summary, inspired by the natural phenomenon that the residual stresses occur naturally in nacre with a brick‐and‐mortar arrangement, we proposed a bioinspired prestressing strategy to enhance the impact‐resistance of nacre‐like structure by working in coordination with tablets sliding toughening mechanism. Synergistic effects between the prestress‐enhanced tablet sliding and prestress‐weakened structural integrality result in an optimized impact resistance at a critical prestress in nacre‐like structures. Impact tests on 3D‐printed nacre‐like specimens with prestress demonstrated proof of concept that the prestressing strategy can work synergistically with the tablets sliding mechanisms at the critical prestress. Finally the bioinspired prestressing strategy was successfully implemented into our designed nacre‐like separators to significantly improve the impact tolerance and electrochemical performances of lithium batteries. Our works revealed the key role of prestress in the impact resistance of nacre specifically, but the proposed prestressing strategy can be tailored and adopted to enhance impact performances of other bioinspired structural materials with heterogeneous architectures. Meanwhile, the bioinspired prestressing strategies combined with tablets sliding mechanisms are inexpensive and can be easily implemented to effectively enhance impact resistance of protective structural and functional materials.

## 4 Experimental Section

Experimental Section details can be found in the Supporting Information.

## Conflict of Interest

The authors declare no conflict of interest.

## Author Contributions

K.J.W. and Y.H.S. contributed equally to this work. Y.N. conceived and guided the research. K.J.W. performed theoretical analyses. K.J.W., Y.H.S., S.S.Z., X.L.G., and H.‐B.Y. performed the synthesis, mechanical testing, and characterizations. K.J.W., L.H.H., H.‐B.Y., and Y.N. analyzed the data and cowrote the manuscript. All authors contributed to the data discussion.

## Supporting information

Supporting Information

## Data Availability

The data that support the findings of this study are available from the corresponding author upon reasonable request.

## References

[1] C. Ortiz , M. C. Boyce , Science 2008, 319, 1053. 18292331 10.1126/science.1154295

[2] U. G. Wegst , H. Bai , E. Saiz , A. P. Tomsia , R. O. Ritchie , Nat. Mater. 2015, 14, 23. 25344782 10.1038/nmat4089

[3] M. Eder , S. Amini , P. Fratzl , Science 2018, 362, 543. 30385570 10.1126/science.aat8297

[4] L. B. Mao , H. L. Gao , H. B. Yao , L. Liu , H. Cölfen , G. Liu , S. M. Chen , S. K. Li , Y. X. Yan , Y. Y. Liu , S. H. Yu , Science 2016, 354, 107. 27540008 10.1126/science.aaf8991

[5] G. Mayer , Science 2005, 310, 1144. 16293751 10.1126/science.1116994

[6] W. Huang , D. Restrepo , J. Y. Jung , F. Y. Su , Z. Liu , R. O. Ritchie , J. McKittrick , P. Zavattieri , D. Kisailus , Adv. Mater. 2019, 31, 1901561. 10.1002/adma.201901561 31268207

[7] R. O. Ritchie , Nat. Mater. 2011, 10, 817. 22020005 10.1038/nmat3115

[8] K. Wu , Z. Song , S. Zhang , Y. Ni , S. Cai , X. Gong , L. He , S. H. Yu , Proc. Natl. Acad. Sci. USA 2020, 117, 15465. 32571926 10.1073/pnas.2000639117 PMC7355047

[9] L. S. Dimas , G. H. Bratzel , I. Eylon , M. J. Buehler , Adv. Funct. Mater. 2013, 23, 4629.

[10] G. X. Gu , M. Takaffoli , M. J. Buehler , Adv. Mater. 2017, 29, 1700060. 10.1002/adma.201700060 28556257

[11] K. A. DeRocher , P. J. Smeets , B. H. Goodge , M. J. Zachman , P. V. Balachandran , L. Stegbauer , M. J. Cohen , L. M. Gordon , J. M. Rondinelli , L. F. Kourkoutis , D. Joester , Nature 2020, 583, 66. 32612224 10.1038/s41586-020-2433-3 PMC8290891

[12] I. Polishchuk , A. A. Bracha , L. Bloch , D. Levy , S. Kozachkevich , Y. Etingergeller , Y. Kauffmann , M. Burghammer , C. Giacobbe , J. Villanova , G. Hendler , C. Y. Sun , A. J. Giuffre , M. A. Marcus , L. Kundanati , P. Zaslansky , N. M. Pugno , P. U. P. A. Gilbert , A. Katsman , B. Pokroy , Science 2017, 358, 1294. 29217569 10.1126/science.aaj2156

[13] E. Seknazi , B. Pokroy , Adv. Mater. 2018, 30, 1707263. 10.1002/adma.201707263 29766594

[14] H. C. Loh , T. Divoux , B. Gludovatz , P. U. Gilbert , R. O. Ritchie , F. J. Ulm , A. Masic , Commun. Mater. 2020, 1, 77.

[15] J. B. Forien , C. Fleck , P. Cloetens , G. Duda , P. Fratzl , E. Zolotoyabko , P. Zaslansky , Nano Lett. 2015, 15, 3729. 26009930 10.1021/acs.nanolett.5b00143

[16] S. J. Eichhorn , D. J. Scurr , P. M. Mummery , M. Golshan , S. P. Thompson , R. J. Cernik , J. Mater. Chem. 2005, 15, 947.

[17] J. Liu , Z. Huang , Z. Pan , Q. Wei , X. Li , Y. Qi , Phys. Rev. Lett. 2017, 118, 105501. 28339258 10.1103/PhysRevLett.118.105501

[18] J. Currey , J. Taylor , J. Zool. 1974, 173, 395.

[19] F. Barthelat , Science 2016, 354, 32. 27846481 10.1126/science.aah6507

[20] H. L. Gao , S. M. Chen , L. B. Mao , Z. Q. Song , H. B. Yao , H. Cölfen , X. S. Luo , F. Zhang , Z. Pan , Y. F. Meng , Y. Ni , S. H. Yu , Nat. Commun. 2017, 8, 287. 28821851 10.1038/s41467-017-00392-z PMC5562756

[21] S. A. Wainwright , Nature 1969, 224, 777.

[22] B. Pokroy , V. Demensky , E. Zolotoyabko , Adv. Funct. Mater. 2009, 19, 1054.

[23] B. Pokroy , V. Demensky , E. Zolotoyabko , Metall. Mater. Trans. A 2011, 42, 554.

[24] V. Schöppler , I. Zlotnikov , The role of residual stresses in biomineral morphogenesis revealed by 3D dark‐field x‐ray microscopy, Bulletin of the American Physical Society, 2020, 65, Abstract: J22.00006.

[25] J. C. Weaver , G. W. Milliron , A. Miserez , K. Evans‐Lutterodt , S. Herrera , I. Gallana , W. J. Mershon , B. Swanson , P. Zavattieri , E. DiMasi , D. Kisailus , Science 2012, 336, 1275. 22679090 10.1126/science.1218764

[26] A. Jackson , J. F. Vincent , R. Turner , Proc. R. Soc. London, Ser. B 1988, 234, 415.

[27] F. Barthelat , H. Tang , P. D. Zavattieri , C. M. Li , H. D. Espinosa , J. Mech. Phys. Solids 2007, 55, 306.

[28] H. Gao , B. Ji , I. L. Jäger , E. Arzt , P. Fratzl , Proc. Natl. Acad. Sci. USA 2003, 100, 5597. 12732735 10.1073/pnas.0631609100 PMC156246

[29] Y. Ni , Z. Song , H. Jiang , S. H. Yu , L. He , J. Mech. Phys. Solids 2015, 81, 41.

[30] B. L. Smith , T. E. Schäffer , M. Viani , J. B. Thompson , N. A. Frederick , J. Kindt , A. Belcher , G. D. Stucky , D. E. Morse , P. K. Hansma , Nature 1999, 399, 761.

[31] J. Liu , W. Zhu , Z. Yu , X. Wei , Acta Biomater. 2018, 74, 270. 29723702 10.1016/j.actbio.2018.04.031

[32] Z. Yin , F. Hannard , F. Barthelat , Science 2019, 364, 1260. 31249053 10.1126/science.aaw8988

[33] K. Wu , Z. Zheng , S. Zhang , L. He , H. Yao , X. Gong , Y. Ni , Mater. Des. 2019, 163, 107532.

[34] G. X. Gu , M. Takaffoli , A. J. Hsieh , M. J. Buehler , Extreme Mech. Lett. 2016, 9, 317.

[35] A. E. Naaman , Prestressed Concrete Analysis and Design: Fundamentals, McGraw‐Hill, New York 1982.

[36] M. Robb , W. Arnold , I. Marshall , Compos. Struct. 1995, 32, 141.

[37] B. Whittingham , I. H. Marshall , T. Mitrevski , R. Jones , Compos. Struct. 2004, 66, 685.

[38] C. Sun , S. Chattopadhyay , J. Appl. Mech. 1975, 42, 693.

[39] S. Khalili , R. Mittal , N. M. Panah , Compos. Struct. 2007, 77, 263.

[40] J. Suhr , N. Koratkar , J. Nanosci. Nanotechnol. 2006, 6, 483. 16573048 10.1166/jnn.2006.096

[41] N. A. Koratkar , J. Suhr , A. Joshi , R. S. Kane , L. S. Schadler , P. M. Ajayan , S. Bartolucci , Appl. Phys. Lett. 2005, 87, 063102.

[42] X. Li , J. Wang , J. Du , M. Cao , K. Liu , Q. Li , X. Q. Feng , L. Jiang , Adv. Mater. Interfaces 2015, 2, 1500250.

[43] Y. H. Song , K. J. Wu , T. W. Zhang , L. L. Lu , Y. Guan , F. Zhou , X. X. Wang , Y. C. Yin , Y. H. Tan , F. Li , T. Tian , Y. Ni , H. B. Yao , S. H. Yu , Adv. Mater. 2019, 31, 1905711. 10.1002/adma.201905711 31693256

[44] A. Kwade , W. Haselrieder , R. Leithoff , A. Modlinger , F. Dietrich , K. Droeder , Nat. Energy 2018, 3, 290.

