# Supplementary material for: A Prestressing Strategy Enabled Synergistic Energy‐Dissipation in Impact‐Resistant Nacre‐Like Structures
Source: Adv Sci (Weinh). 2022 Jan 12;9(6):2104867. doi: 10.1002/advs.202104867 (PMC8867135; doi:10.1002/advs.202104867)
Supplement: Supplementary file 1 — Supporting Information [file ADVS-9-2104867-s001.pdf]

ADVANCED SCIENCE Open Access

Supporting Information

for *Adv. Sci.*, DOI: 10.1002/advs.202104867

A Prestressing Strategy Enabled Synergistic Energy-dissipation in Impact-resistant Nacre-like Structures

Kaijin Wu[1], Yonghui Song[2], Xiao Zhang[1], Shuaishuai Zhang[1], Zhijun Zheng[1], Xinglong Gong[1], Linghui He[1], Hong-Bin Yao[2], Yong Ni[1]**

Supporting Information

A Prestressing Strategy Enabled Synergistic Energy-dissipation in Impact-resistant Nacre-like Structures

Kaijin Wu[1†], Yonghui Song[2†], Xiao Zhang[1], Shuaishuai Zhang[1], Zhijun Zheng[1], Xinglong Gong[1], Linghui He[1], Hong-Bin Yao[2]*, Yong Ni[1]*

[1] *CAS Key Laboratory of Mechanical Behavior and Design of Materials, Department of Modern Mechanics,* CAS Center for Excellence in Complex System Mechanics, *University of Science and Technology of China, Hefei, Anhui 230026, China*

[2]*Division of Nanomaterials & Chemistry, Hefei National Laboratory for Physical Sciences at the Microscale, Department of Chemistry, Institute of Biomimetic Materials & Chemistry, University of Science and Technology of China, Hefei, Anhui 230026, China*

†These authors contributed equally to this work.

*Correspondence and requests for materials should be addressed to the author:

Hong-Bin Yao (yhb@ustc.edu.cn), Yong Ni (yni@ustc.edu.cn).

Experimental Section

Modeling of impact responses: In order to demonstrate the effects of residual prestress on the impact responses of nacre-like structure, a three-dimensional nonlinear finite element model (FEM) of brick-and-mortar structures mimicking natural nacre was developed using the commercial software ABAQUS v6.14 (**Figure S1**a). The nacre-like structure comprises five layers of stiff bricks, and each

layer ($60 \times 60 \times 0.2$ mm^3) made of almost 120 random polygonal tablets mimicking natural nacre was generated through Voronoi technology.[1,2] It's worth mentioning that although the absolute sizes of the nacre-like model we built here are different from the natural nacre, the architectural features of brick-and-mortar arrangements are consistent with natural nacre, where aspect ratio of the tablets in FEM is about 30 and the overlap areas between tablets from adjacent column cover about 1/3 of the surface area of the tablet layers.[3] The nacre-like polygonal tablets was meshed by using ABAQUS solid elements (C3D8R and C3D6R) with size about 0.1 mm. There are two layers of element mesh in the one brick layer to capture the bending effects of plate. The adjacent stiff lamellae are separated by soft mortar layer with negligible thickness, and the soft mortar layers were meshed using ABAQUS solid elements (COH3D8). A cylindrical impactor with 10 mm in diameter and 0.1 kg in mass is treated as a rigid body and is fully constrained except in the loading direction. Our simulations focused mainly on the effects of prestress on impact-resistance, thus we chose a representative impacting speed of 2.5 m/s. Under this impact velocity, natural nacre can sustain the strike of mantis shrimps through tablets sliding.[4] Two rigid frames with 5 mm width are constructed to clamp the nacre-like model. The prestressing effect is applied by predefining stress field in ABAQUS. In this work, we changed the values of predefining stress field to analyze the effects of prestress on impact-resistance of nacre-like structure. For low-velocity impaction, our simulations focused mainly on the impact energy dissipation dominated by competitive prestress-controlled failure modes, such as radial cracks propagation and tablets sliding, thus we neglected the strain rate effects of materials to simplify our models, and the effects of the strain rate effects of materials on impact-resistance of nacre can refer to previous works.[6] The stiff bricks with the failure strength of 200 MPa, isotropic bulk modulus of 100 GPa, Poison ratio of 0.33 and density of 3 g(cm)$^{-3}$ were simulated using brittle cracking model in ABAQUS. The soft mortars with the failure strength of 25 MPa, shear modulus of 0.8 GPa and

critical energy release rate of 10.61 J/m^2 were modeled by cohesive elements with a trapezoidal cohesive law, which represents ideal elastic-plastic nonlinear behaviors due to tablets sliding and polymer elongation in natural nacre (Figure S1b and Figure S1c). Experimental investigations obtained by Smith et al. show that some of the molecules present in the interface exhibit large stretching capability and an approximately constant strength over the range of displacements (Figure S1b).[6] Therefore, it seemed reasonable to assume a constant shear strength for the cohesive law without considering the possibility of polymer (interface) stiffening at larger strains, and details of a typical cohesive zone model with trapezoidal cohesive law is shown in Figure S1c. The quadratic form stress was adopted for damage initiation criterion:

$$\left(\frac{\langle\sigma\rangle}{\sigma_0}\right)^2+\left(\frac{\tau}{\tau_f}\right)^2=1 \tag{1}$$

where < > is the Macaulay bracket, σ and τ are tensile stress and shear stress, and we assumed the mixed-mode behavior was mode-independent, thus the interfacial tensile strength (σ_0) and shear strength (τ_f) are taken as 25 MPa.

After the damage initiation, the cohesive traction remains constant at maximum stress between certain separations followed by softening and failure. The trapezoidal shaped damage evolution was defined by tabular softening in ABAQUS. The softening response of cohesive element is defined as follows：

$$T_i=(1-D)K_i\delta_i \tag{2}$$

where D is scalar stiffness degradation (SDEG in ABAQUS), D = 0 represents that interface is undamaged, and D = 1 represents the interface is fully fractured. For the trapezoidal cohesive law, the damage evolution variable D can be directly defined as a tabular function of the effective displacement as follow:

$$D = \begin{cases} 0, & 0 \le \delta \le \delta_1 \\ 1 - \dfrac{\delta_1}{\delta}, & \delta_1 \le \delta \le \delta_2 \\ 1 - \dfrac{\delta_1}{\delta}\dfrac{\delta_3 - \delta}{\delta_3 - \delta_2}, & \delta_2 \le \delta \le \delta_3 \\ 1, & \delta \ge \delta_3 \end{cases} \tag{3}$$

where δ is separation displacement and details for δ_1 to δ_3 of nacre can be seen in **Table S1**.

Further, **Figure S9**a shows the 3D nonlinear finite element models for the nacre-inspired separator (NS) and the commercial separators (CS) under ball impact. In simulations, the nacre-like coating is made of ten 12 cm × 12 cm × 20 μm-thick layers. The polyethylene (PE) layer with size 12 cm × 12 cm × 0.4 mm is mesh using solid element C3D8R. The commercial nanoparticles coating is modeled as a bulk structure with thickness about 0.2 mm similar to that of the nacre-like coating. The sphere impactor with 3 cm in diameter and 65 g in mass is regarded as rigid body. The PE layer with bulk modulus E = 150 MPa, Poison ratio v = 0.3, and density ρ = 0.7 g/cm^3. The commercial ceramic nanoparticles coating with bulk modulus E = 300 GPa, Poison ratio v = 0.3, density ρ = 3 g/cm3 and the failure strength σ_f = 200 MPa before brittle failure. In simulations, the rigid ball has initial impact velocity 3.5 m/s and the supports are full fixed, which are consistent with experiments.

3D-printing fabrication of nacre-like composite: Nacre-like composites composed of stiff bricks and soft mortars were fabricated via additive manufacturing using an Object260 Connex 3 multi-material printer (Stratasys Ltd.). The two constituent materials are a rigid polymer VeroWhitePlus with a Young's modulus of 0.8 GPa and a rubbery polymer TangoblackPlus with a Young's modulus of 0.2 MPa. The high modulus contrast between the hard phases and soft phases (about 4000) is consistent with that of nacre, where the hard mineral bricks has Young's modulus of 100 GPa and the soft mortars has Young's modulus of 30-60 MPa in nacre.[7] The dogbone tensile specimens and three-points

bending specimens were printed to characterize the mechanical properties of the polymers. The tensile specimen is characterized by a gauge dimension of 40 mm, a width of 5 mm and a thickness of 2 mm (**Figure S3**a and Figure S3b). A "rail-shear" dogbone specimen was printed to characterize the shear performances of TangoblackPlus (Figure S3c). The geometry of the rail is characterized by a length of 9 mm, a width of 3 mm and a thickness of 0.1 mm. The printer can print multiple materials simultaneously through a multi-material jetting technology and has a print precision of 16 μm in the layer deposition direction and 600 dpi in the print plane. Excellent adhesion between different materials can be achieved based on an *in situ* curing process. The designed nacre-like composites with thickness of 5 mm have a square impact zone with side length of 45 mm, four fixed sides with width of 8 mm and designed holes, which can be used for droptower impact tests and applying prestress. The arrangements of random polygonal tablets mimicking natural nacre was generated through Voronoi technology (**Figure S4**a), where 200 nuclei are generated in each layer with side length of 60 mm and the numbers of random seed for insertion from first layer to fifth layer are 20, 40, 60, 80 and 100. The random seed for insertion can form random overlaps between adjacent layers and the thickness of vertical interface between intralayer adjacent platelets is about 0.1 mm (Figure S4b). Further, the 3D models for 3D-printed nacreous structure were constructed using the commercial software Solidworks.

Droptower impact tests under pre-tensioning: The characteristics of low-velocity impact resistance for the 3D-printed nacre-like composites under prestress were investigated using a droptower testing machine (MTS Industrial Systems) (**Figure S5**). The specimens with designed holes on sides were held fixed in the fixture of a designed pretension device, leaving square specimens with a side length of 45 mm exposed to the impactor. The designed small pretension device can apply pretensile forces and

measure the values of forces by internal strain gauge before impact. After applying pretensile force, the hemispherical impactor with a mass of 2.16 kg and a diameter of 10 mm was dropped from an initial height h_0 = 315 mm to perform the impaction tests. The initial impact velocity was calculated through the equation $V_0 = \sqrt{2gh_0}$ (*g* is the standard gravity) and the velocity during the impact process is $V(t) = V_0 + \int_0^t a(\tau)d\tau$, where $a(t)$ is the acceleration, which can be collected by a piezoelectric acceleration sensor. The displacement *U* of the impactor is $U(t) = \int_0^t V(\tau)d\tau$, and the contact force *F* was calculated $F = ma$ as based on Newton second law. The energy dissipation E_d is defined as the area under the contact force-displacement curves. The failure patterns are observed from the face opposite the impact and the dynamics is recorded with a high speed camera. The X-ray CT were performed on the specimens recovered from the fixture after impaction to construct damage zone morphologies. The area of the damage zone are calculated by the software ImageJ.

Preparation of a nacre-like separator:[8] The PAP slurry was obtained by mixing PAP (20 wt.%) with an ethanol/deionized water (volume ratio 1:1) solution containing carboxymethylcellulose sodium (CMC, Aladdin) (2 wt.%), where the detailed preparation of PAPs can be found in our previous work.[8] The obtained slurry was slowly stirred until the PAP were uniformly mixed with the CMC binder. The obtained slurry was coated on both sides of a 9 μm thick PE separator and then dried at 60 °C for 24 hours to remove ethanol and deionized water. The PAP can form the nacre-like multilayer structure on the surface of the PE under the assistance of shear stress generated by the blade and solvent evaporation induced self-assembly, and this coating method can be applied in a large scale to satisfy current battery industry process.

Ball impact tests of separators with prestress: The specimens with designed holes on sides were held

fixed in the fixture of a designed pretension device, leaving square specimens with a side length of 45 mm exposed to the impactor (**Figure S7**). The designed small pretension device can apply pretensile forces and measure the values of forces by internal strain gauge before impact. After applying pretensile forces, a steel ball with 65 g in mass and 2.5 cm in diameter was falling freely from a height of 60 cm.

Electrochemical Performances Tests: Symmetric cells were assembled by using Li metal foils as the electrodes and 1 M $LiPF_6$ in EC/DMC/DEC (1:1:1, v/v/v) as the electrolyte. The LiFePO4 cathode was obtained from $LiFePO_4$ powder (MTI Kejing Group), super P carbon black and PVDF ($LiFePO_4$ /carbon black/PVDF 80/10/10, w/w/w). A $LiFePO_4$/Li coin cell was assembled by sandwiching separator between a $LiFePO_4$ cathode and a Li metal foil, and then filling with the 100 μL of the liquid electrolyte of 1 M $LiPF_6$ in EC/DMC/DEC (1:1:1, v/v/v). $LiFePO_4$ cathode was fabricated on the Al foil with an aerial capacity of ~ 1.02 mAh cm^{-2}. The cell operating temperature was 25 ^{o}C. The electrochemical performance test of the $LiFePO_4$/Li coin cell was carried out on a Land multichannel test system under a constant current charge and discharge mode between 2.5 V-4.0 V (vs. Li+/Li).

Characterizations: Scanning electron microscope (SEM, JEOL-6700F) were used to observe the cross-section morphologies of the Abalone shell and the impacted separators. X-ray computed tomography (X-ray CT, Dage Quadra 7) was used to analyse the three-dimensional failure morphologies of impacted 3D-prined nacre-like specimens.

Table S1. Material parameters of the trapezoidal cohesive law[3]

T_c	δ_1	δ_2	δ_3	G_c
25 MPa	0.9 nm	250 nm	600 nm	10.61 J m^{-2}

Supplementary Figures

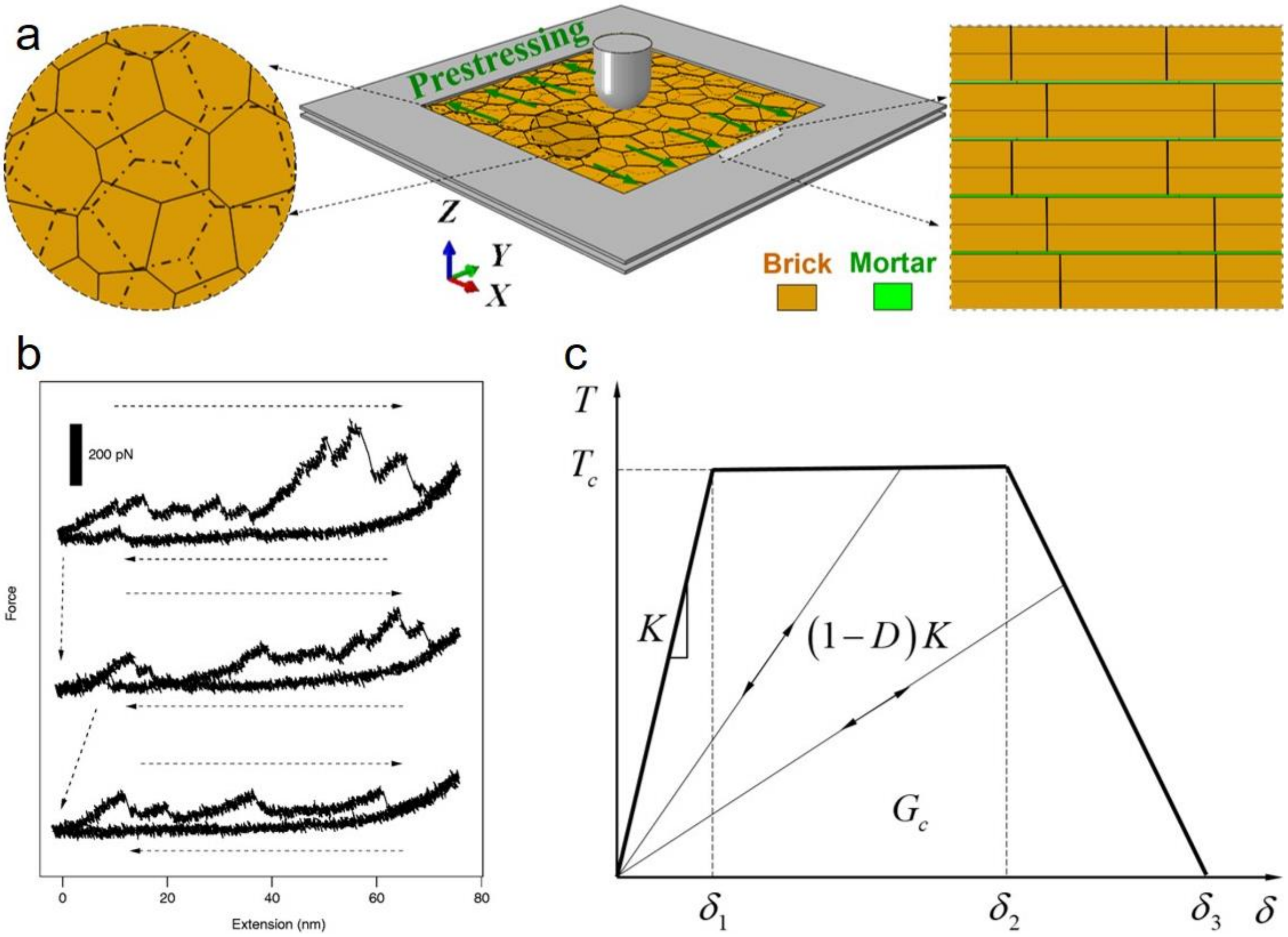

Figure S1. Finite element analyses for impact performances of nacre-like structure with prestress. a) Three-dimensional finite element model of the nacre-like structure with prestress under impact loading, where top view shows Voronoi diagrams mimicking the arrangement of tablets in natural nacre, and the side view shows "brick-and-mortar" staggered arrangement. b) Experimental investigations of natural adhesives elongation (3). c) Schematic cohesive zone model with a

trapezoidal cohesive law.

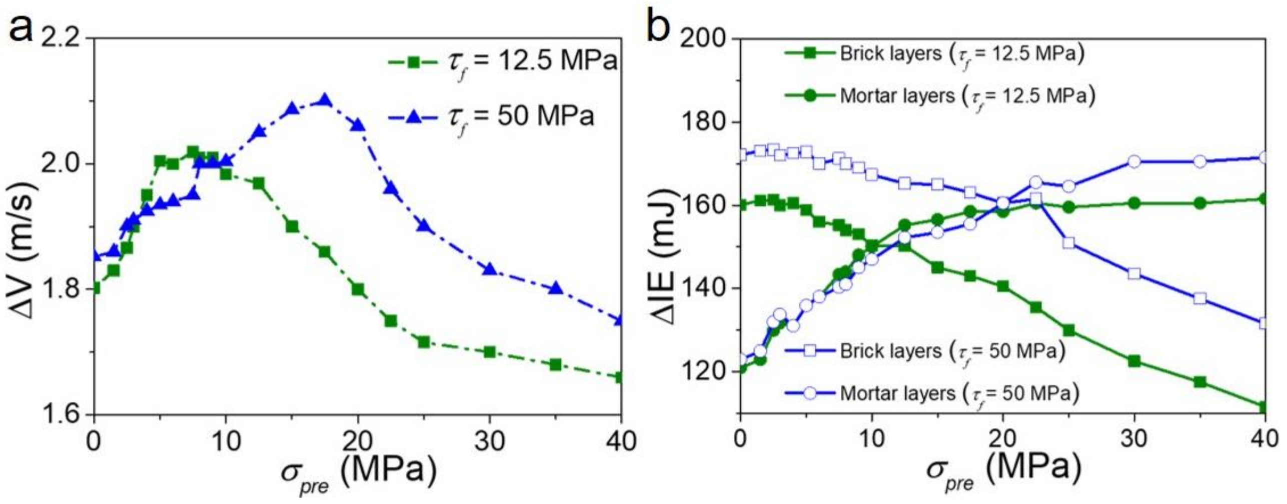

Figure S2. Effects of interfacial strength on the critical prestress. a) Impact speed loss ΔV - prestress σ_{pre} curves for nacre-like structures with different interfacial strengths τ_f. b) The internal energy dissipations ΔIE in mortar layers and brick layers of nacre-like structures with different τ_f under different σ_{pre}.

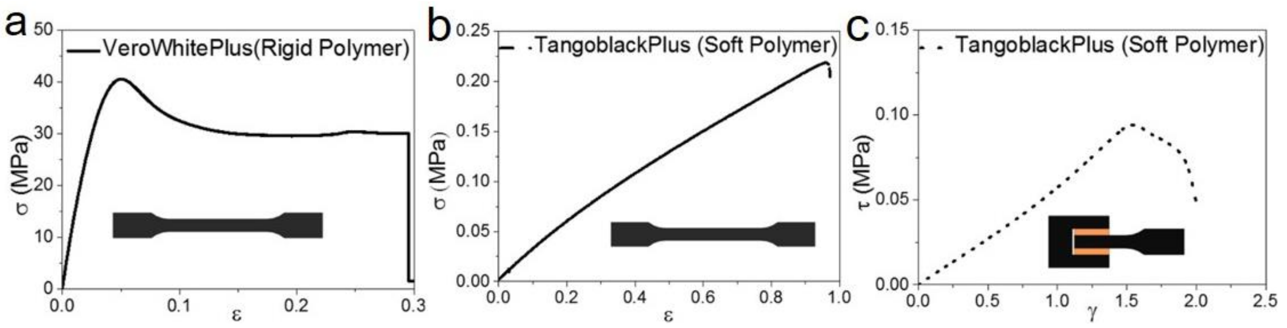

Figure S3. Properties of composite constituents in 3D printing. a-c) Representative stress-strain curves for (a) uniaxial tensile tests on pure stiff brick phases, (b) uniaxial tensile tests on pure soft mortar phases and (c) Simple shear test on pure soft mortar phase.

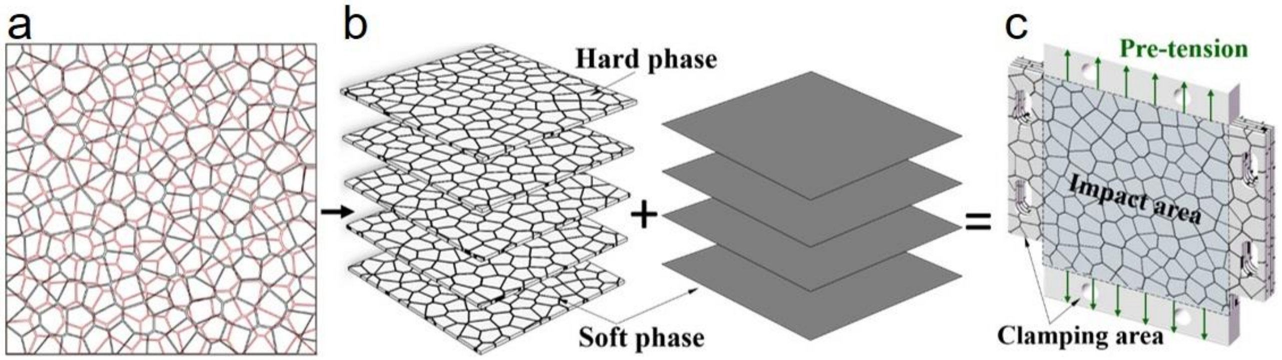

Figure S4. Geometry set-up of the 3D-printed nacre-like structures in impact tests. a) Design of the tablets arrangements from one layer to the next, which mimics the arrangement of tablets in natural nacre (4). b) Design of the nacre-like structure with five brick layers and four mortar layers. c) Representative 3D-printed nacre-like specimen configuration used for drop-tower impact tests under pre-tensioning.

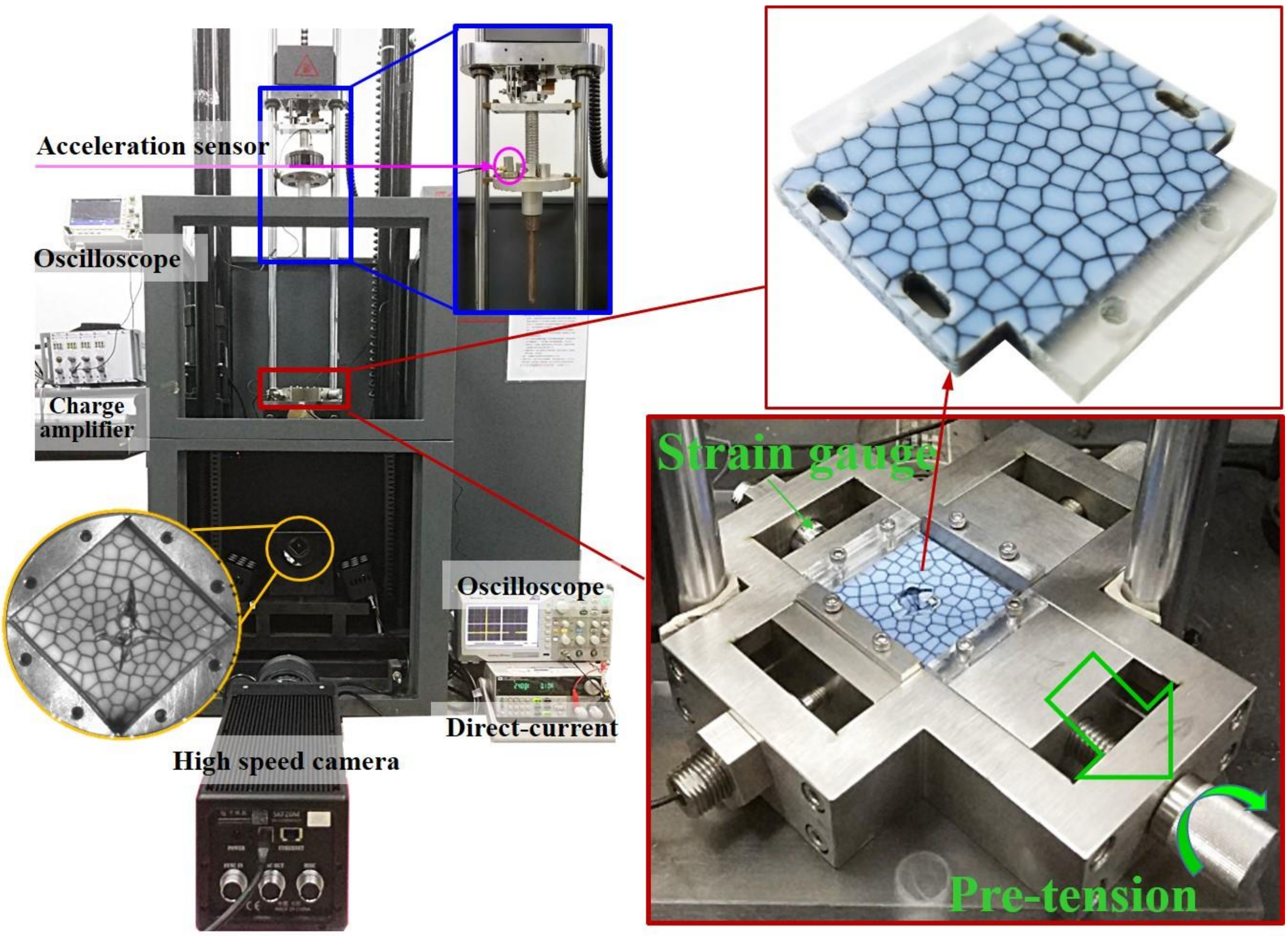

Figure S5. Experimental setup of the drop-tower tests for the 3D-printed nacre-like structures with

prestresses. Drop-tower impact testing machine equipped with a high speed camera. Right insets show a 3D-printed nacre-like specimen and a designed pretensile device.

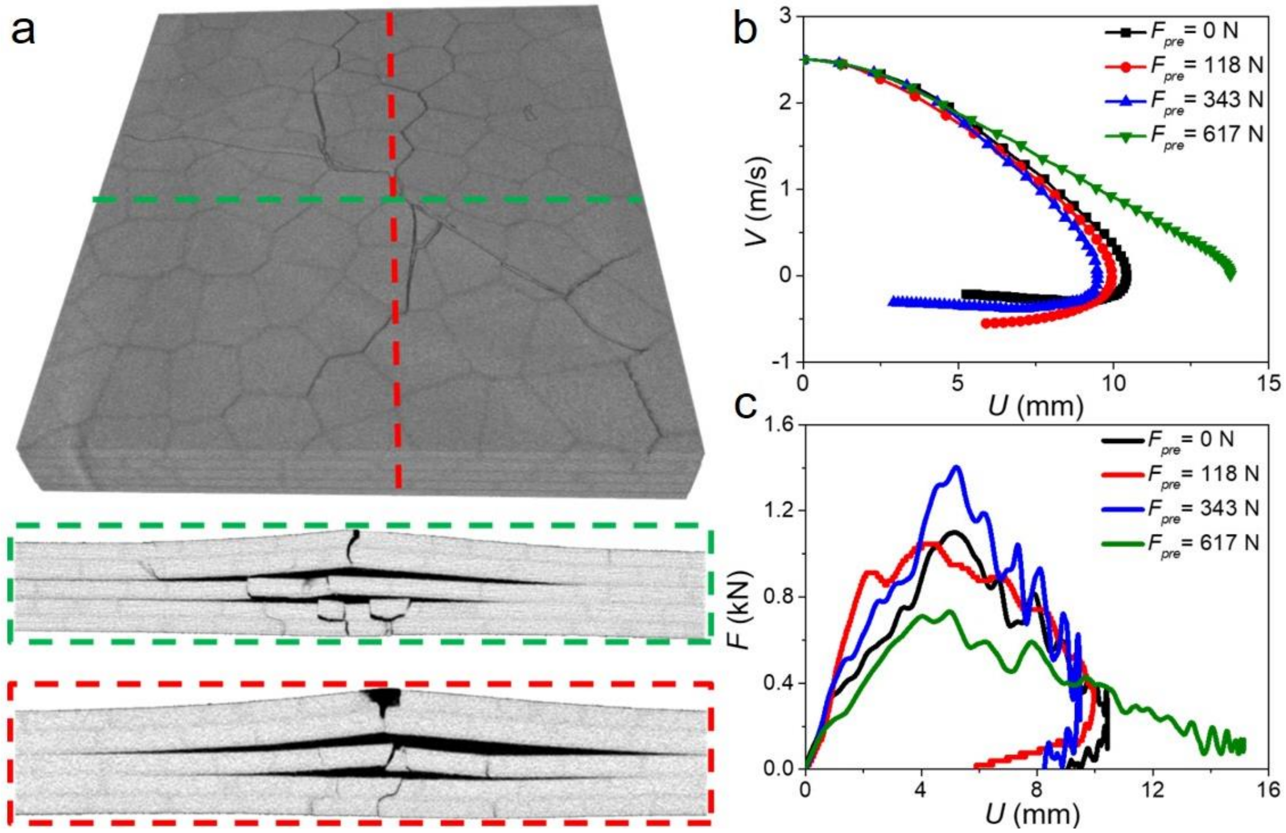

Figure S6. Impact responses of 3D-printed nacre-like specimens with prestress. a) Three-dimensional X-ray CT reconstruction and cross-section images of the impacted nacre-like samples. b) Experimental residual velocity V and displacement U curves under different pretensile forces F_{pre}. c) Contact force F and displacement U curves under different F_{pre}.

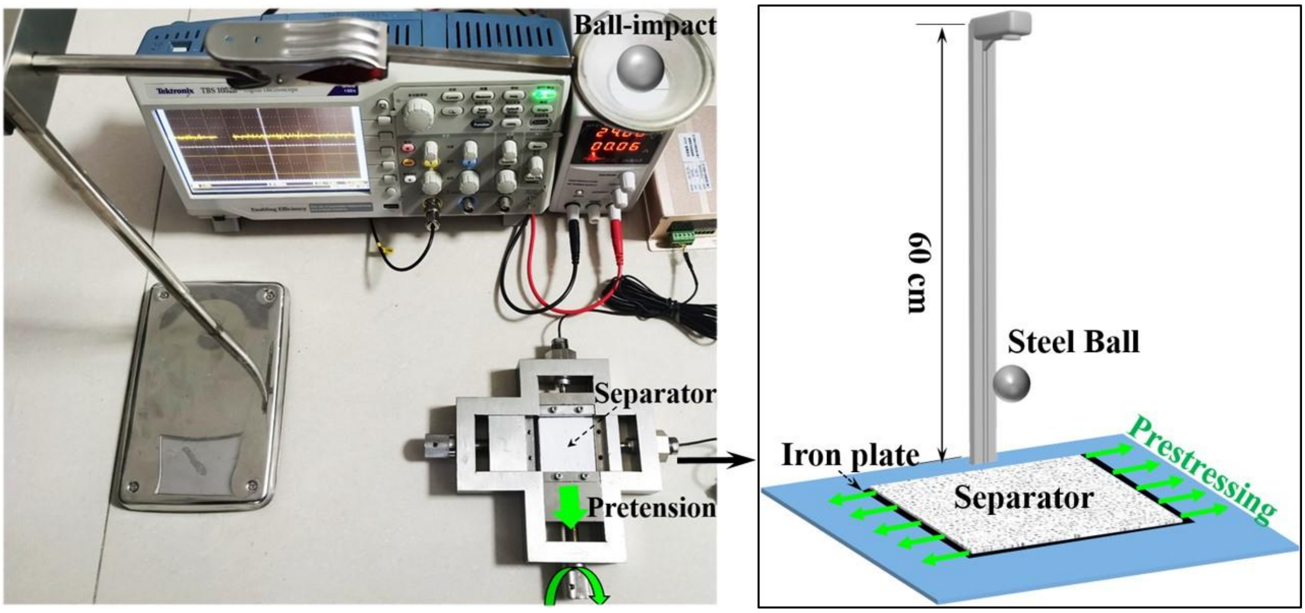

Figure S7. Ball impact tests of separators under pre-tensioning. Experimental setup for a designed system used for ball impact tests of separators with prestressing.

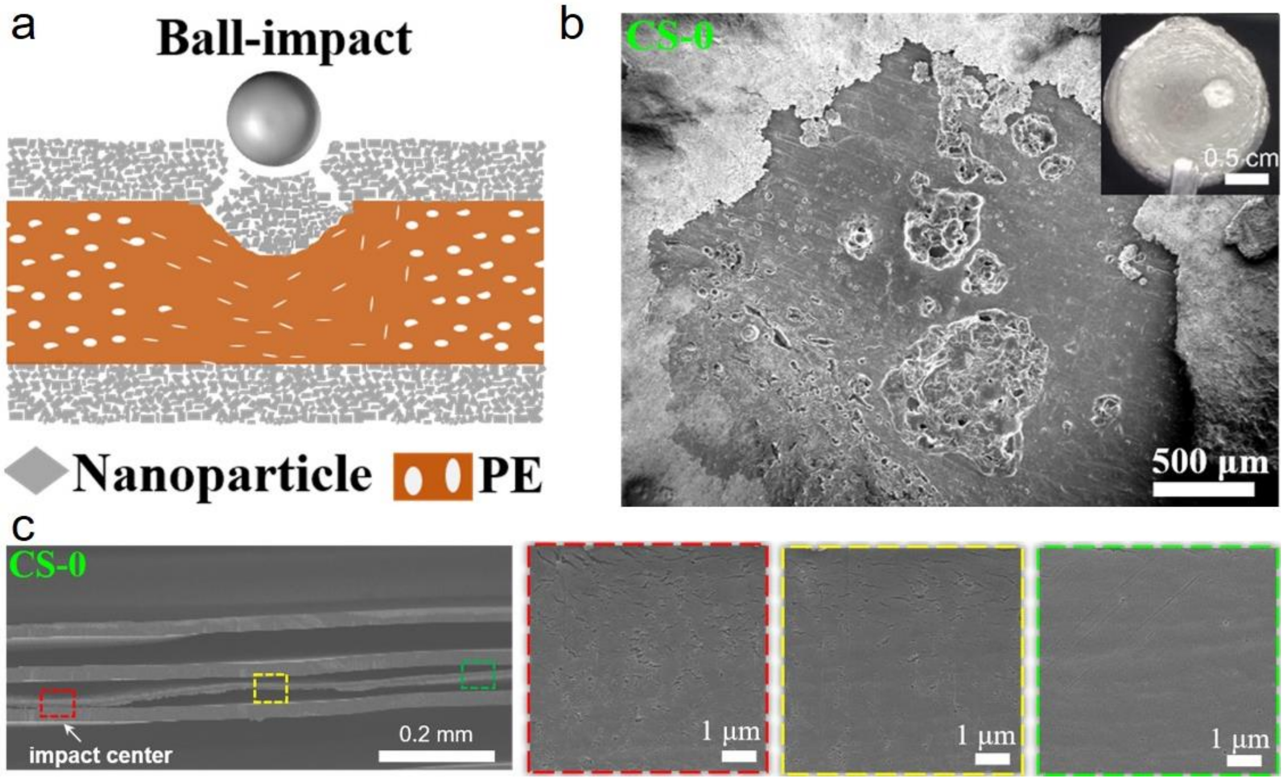

Figure S8. Impact performances of commercial separators for lithium batteries. a) Schematic impact-resistance of commercial separators (CS) composed of ceramic nanoparticles coatings and a microporous polyolefin (PE) membrane. b) Top-view SEM images of Li foil after Li plating using

the impacted CS without prestress (CS-0). c) Overview of the cross-sectional SEM image of the impacted CS-0, and different color squares correspond to the enlarged SEM images of different locations.

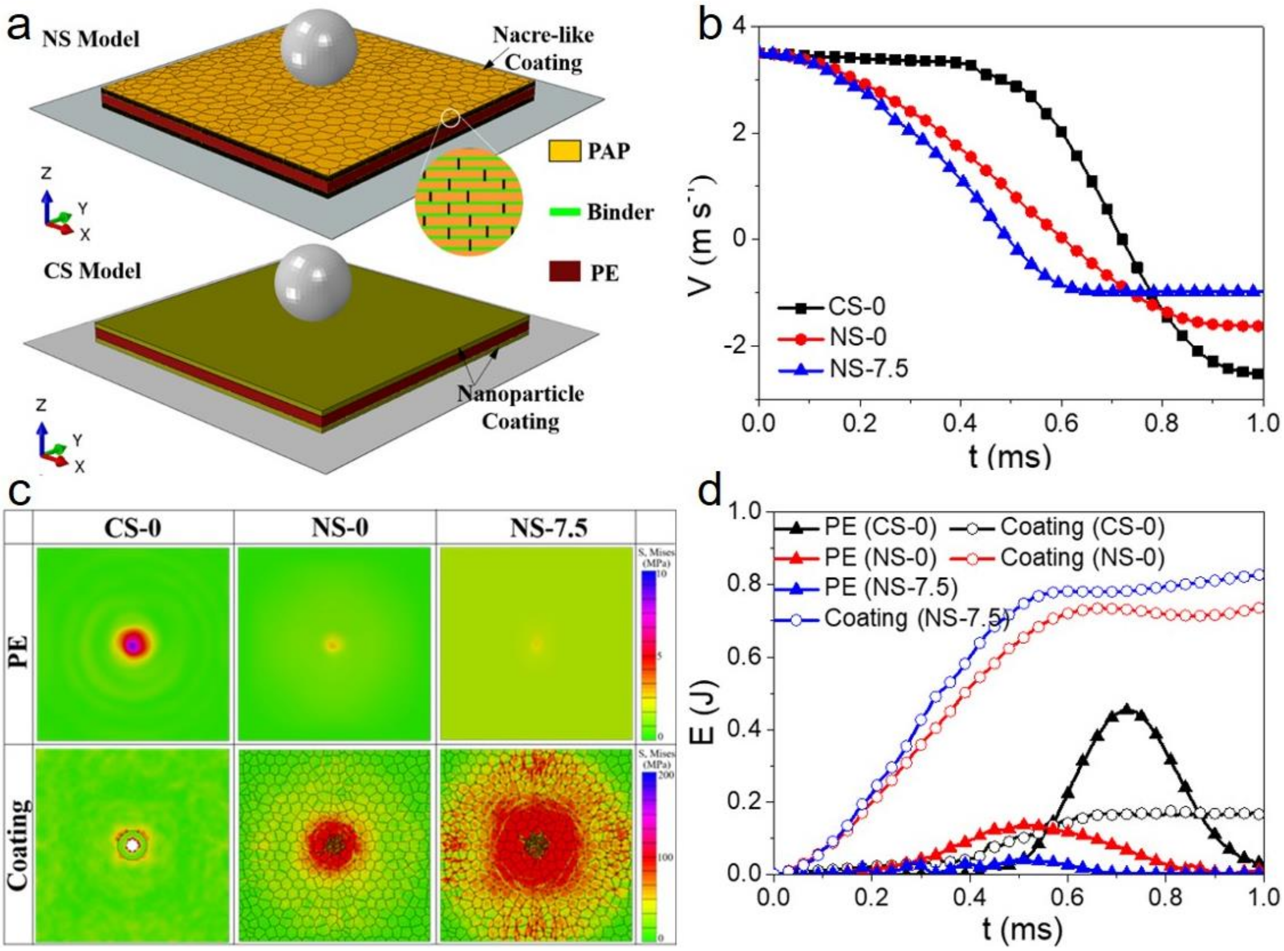

Figure S9. Simulations of impact performances of separators under different prestresses. a) Finite element models of the nacre-inspired separator (NS) and the commercial separators (CS) under ball impacting. b) Residual velocity (V) and time (t) curves for CS-0, NS-0 and NS-7.5, where 0 and 7.5 represent that the pretensile forces are 0 N and 7.5 N respectively. c) Snapshots of von Mises stress fields in the PE layer and coatings of CS-0, NS-0 and NS-7.5. d) Energy dissipations in the PE layer and coatings of CS-0, NS-0 and NS-7.5.

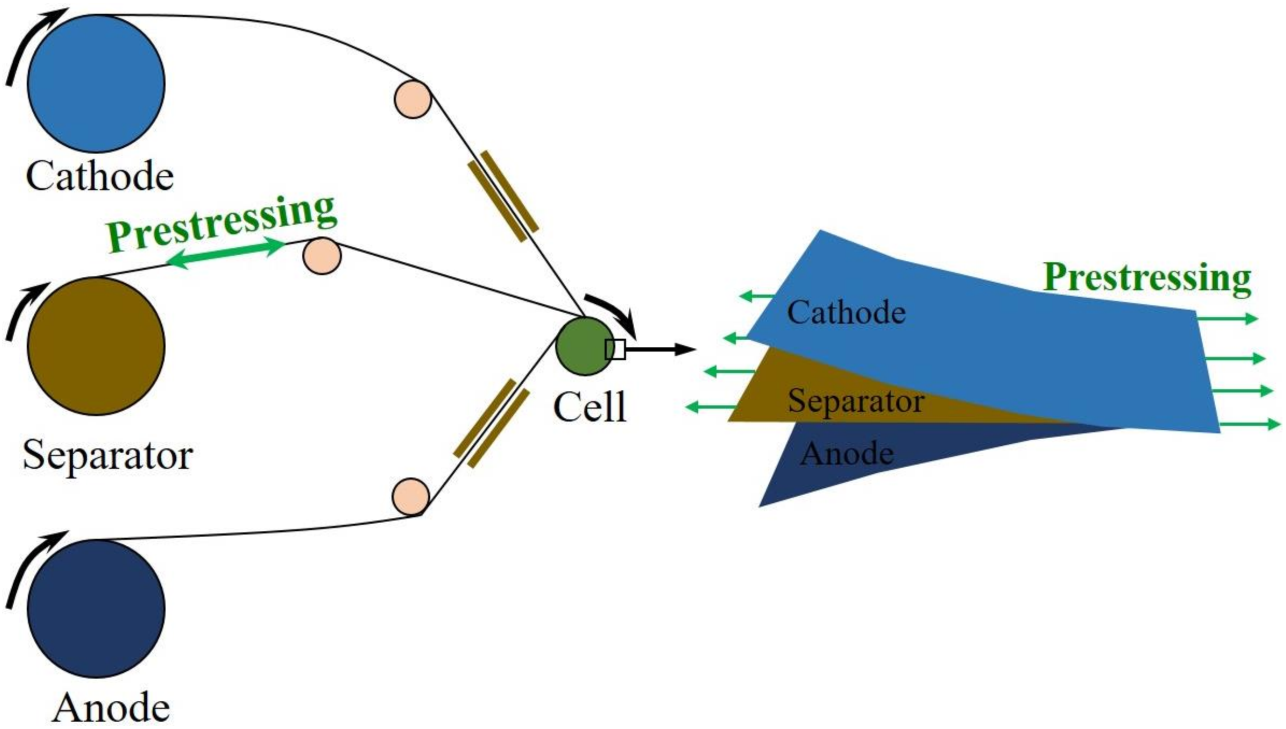

Figure S10. Schematic for one idea of applying prestress on separators during the winding process of lithium-ion battery production.

Supplementary References

[1] A. Ghazlan, T. D. Ngo, P. Tran, *Composite Structures* **2016**, *153*, 278.

[2] Z. Zheng, C. Wang, J. Yu, S. R. Reid, J. J. Harrigan, *Journal of the Mechanics and Physics of Solids* **2014**, *72*, 93.

[3] F. Barthelat, H. Tang, P. D. Zavattieri, C. M. Li, H. D. Espinosa, *Journal of The Mechanics and Physics of Solids* **2007**, *55*, 306.

[4] X. Li, J. Wang, J. Du, M. Cao, K. Liu, Q. Li, X. Q. Feng, L. Jiang, *Advanced Materials Interfaces* **2015**, 2, 1500250.

[5] J. Liu, W. Zhu, Z. Yu, X. Wei, *Acta Biomaterialia* **2018**, *74*, 270.

[6] B. L. Smith, T. E. Schäffer, M. Viani, J. B. Thompson, N. A. Frederick, J. Kindt, A. Belcher, G. D. Stucky, D. E. Morse, P. K. Hansma, *Nature* **1999**, *399*, 761.

[7] H. Gao, B. Ji, I. L. Jäger, E. Arzt, P. Fratzl, *Proceedings of the national Academy of Sciences* **2003**, *100*, 5597.

[8] Y. H. Song, K. J. Wu, T. W. Zhang, L. L. Lu, Y. Guan, F. Zhou, X. X. Wang, Y. C. Yin, Y. H. Tan, F. Li, T. Tian, Y. Ni, H. B. Yao, S. H. Yu, *Advanced Materials* **2019**, *31*, 1905711.
